# Cysteine–based redox regulation and signaling in plants

**DOI:** 10.3389/fpls.2013.00105

**Published:** 2013-04-29

**Authors:** Jérémy Couturier, Kamel Chibani, Jean-Pierre Jacquot, Nicolas Rouhier

**Affiliations:** UMR1136 Université de Lorraine-INRA, Interactions Arbres/Micro-organismes, IFR110, Faculté des SciencesVandoeuvre, France

**Keywords:** cysteine, disulfide bond, glutathionylation, nitrosylation, redox regulation, sulfenic acid, thiolate

## Abstract

Living organisms are subjected to oxidative stress conditions which are characterized by the production of reactive oxygen, nitrogen, and sulfur species. In plants as in other organisms, many of these compounds have a dual function as they damage different types of macromolecules but they also likely fulfil an important role as secondary messengers. Owing to the reactivity of their thiol groups, some protein cysteine residues are particularly prone to oxidation by these molecules. In the past years, besides their recognized catalytic and regulatory functions, the modification of cysteine thiol group was increasingly viewed as either protective or redox signaling mechanisms. The most physiologically relevant reversible redox post-translational modifications (PTMs) are disulfide bonds, sulfenic acids, S-glutathione adducts, S-nitrosothiols and to a lesser extent S-sulfenyl-amides, thiosulfinates and S-persulfides. These redox PTMs are mostly controlled by two oxidoreductase families, thioredoxins and glutaredoxins. This review focuses on recent advances highlighting the variety and physiological roles of these PTMs and the proteomic strategies used for their detection.

## INTRODUCTION

The production of reactive oxygen (ROS) and nitrogen (RNS) species is unavoidable under aerobiosis, especially at the level of electron transfer chain reactions for ROS or via enzymatic processes for RNS. Moreover, their generation is often exacerbated in plants facing abiotic and biotic constraints (Moller et al., 2007). Another category of molecules, called reactive sulfur species (RSS), is currently the subject of intense investigation as the major form, hydrogen sulfide (H_2_S) was shown to have physiological effects in animals and is also encountered by plants either in their environment or from intracellular production (Lisjak et al., 2013). ROS include superoxide radical ($O_{2}^{\cdot -}$), hydrogen peroxide (H_2_O_2_), hydroxyl radical (OH·), and singlet oxygen (^1^O_2_), whereas RNS essentially designate nitric oxide (·NO) and derived molecules such as nitrogen dioxide (NO_2_), dinitrogen trioxide (N_2_O_3_) and peroxynitrite (ONOO^-^). Interestingly, some interplay exists between ROS and RNS as peroxynitrite is formed via a reaction of NO with $O_{2}^{\cdot -}$. Several of these molecules have dual functions since they damage cellular components such as proteins, DNA, and lipids but, at sub-toxic concentrations, they constitute signaling molecules both for plant development, physiology, and immunity (Besson-Bard et al., 2008; Mittler et al., 2011; Schippers et al., 2012; Yu et al., 2012; Lisjak et al., 2013). Hence, it is necessary to tightly regulate their intracellular concentrations. A physiologically relevant signaling molecule should accumulate transiently, being quickly formed and removed at specific cellular microenvironments, and it should be perceived/relayed by specific target proteins (receptors, transcription factors, kinases, phosphatases, or other) through reversible post-translational modifications (PTMs) for downstream intracellular signaling (Vestergaard et al., 2012). Owing to its reactivity and its numerous oxidation states, changes in cysteine oxidation are now recognized as cellular switches modulating biological activity of proteins, thus mediating critical cellular events in response to environmental stimuli, similarly, to phosphorylation cascades.

In this review, we focus on ROS-, RNS- and RSS-mediated oxidations occurring on cysteine thiol groups of selected proteins, referred to as redox PTMs. Besides, we describe the current proteomic methods developed to detect, identify and eventually quantify them.

## MULTIPLE CYSTEINE OXIDATION FORMS FOR DISTINCT REDOX SIGNALS

Due to their unique physico–chemical properties, cysteinyl residues participate in catalytic reactions, serve as metal ligands and are also susceptible to various PTMs. Whereas free cysteines have an ionization constant (pKa) of about 8.3, in proteins, some cysteines defined as reactive cysteines, possess lower pKa, ranging from three to seven. In thiol-oxidoreductases as thioredoxins (Trxs) and glutaredoxins (Grxs), the lowering of the pKa results from the protein microenvironment as these thiolates are stabilized by proximal positively charged amino acids, by specific hydrogen bonding and/or by a dipole effect induced at the N-terminus of an α-helix. This implies that, at physiological pH, these residues will be predominantly found as thiolates, which are much stronger nucleophiles than thiol groups. Consequently, proteins containing these reactive cysteines can undergo many different oxidation states in response to different redox signals. It is for instance not yet clear which protein properties favor one modification vs another but the local protein environment and the proximity to the oxidant source may be important parameters. While cysteines react covalently with lipids or fatty acids undergoing palmitoylation, prenylation or Michael addition to oxidized lipids, we will concentrate on PTMs generated on protein thiol groups via ROS, RNS, and RSS, particularly H_2_O_2_, ·NO, and H_2_S. There is an interesting parallel here because these three molecules, which were initially thought to be exclusively toxic, may in fact represent key regulators for various biological processes and in particular for signaling purposes, considering their relative stability, their capacity to diffuse across membranes (·NO) and H_2_S are gases and the uncharged H_2_O_2_ is channeled via aquaporins) and their propensity to react with thiolates (Bienert et al., 2007).

### H_2_O_2_-MEDIATED MODIFICATION OF PROTEIN THIOLS

**FIGURE 1:**
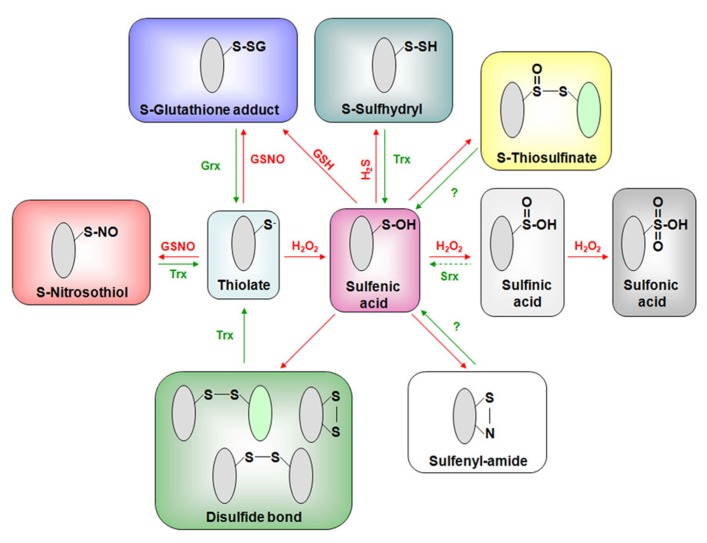
**Principal oxidative modifications of cysteinyl residues and their reduction pathways.** Reactive cysteine residues mostly exist in thiolate forms at physiological pH and can form a sulfenic acid (SOH) by reacting with H_2_O_2_. This sulfenic acid is an intermediate for most other redox PTMs forming (i) intra- or intermolecular disulfide bonds, (ii) glutathione adducts in the presence of GSH, (iii) sulfenyl-amides, (iv) persulfides upon reaction with hydrogen sulfide, (v) thiosulfinates by reacting with another sulfenic acid, and (vi) sulfinic (SO_2_H) and sulfonic (SO_3_H) acids by further reacting with H_2_O_2_. Another possible glutathionylation pathway could rely on the reaction between a thiolate and nitrosoglutathione (GSNO). Interestingly, the latter compound will also promote S-nitrosylation reactions similarly, to some other derived forms of NO not represented here (see the text). Most of these redox PTMs are reversible and the enzymatic reduction of these different oxidation forms is essentially achieved by glutaredoxins (mainly glutathionylated proteins but also some disulfides) and thioredoxins (disulfides and possibly persulfides, nitrosothiols and some glutathionylated proteins). In addition to specifically reducing sulfinic acids formed on peroxiredoxins, sulfiredoxin (Srx) may also catalyze Prx deglutathionylation (Findlay et al., 2006; Park et al., 2009). It is not yet demonstrated that plant Trxs have a denitrosylase activity. The reduction pathway of thiosulfinates, sulfenyl-amides is unclear but glutathione and subsequently Grxs might be involved.

With methionines, cysteines are the most H_2_O_2_-sensitive residues. The two-electron oxidation of a cysteine thiolate (-S^–^) by H_2_O_2_ forms a sulfenic acid (-SOH; **Figure 1**). Due to its unstable and highly reactive nature, the sulfenic acid will be further modified (Reddie and Carroll, 2008). In the absence of other proximal thiolates, sulfenic acid can further react with one or two additional peroxides, forming sulfinic (-SO_2_H) and sulfonic (-SO_3_H) acids (**Figure 1**). These two modifications are usually considered as irreversible reactions, except for the SO_2_H in the specific case of the peroxiredoxin (Prx) catalytic cycle. Alternatively, sulfenic acids can react with the main chain nitrogen of a neighboring residue to form a sulfenyl-amide or condensate with another sulfenic acid leading to thiosulfinate but the physiological relevance of these modifications is unclear (**Figure 1**). Most of the time, the sulfenic acid will react with another thiolate from a protein cysteine or glutathione, leading to the formation of an intra- or inter-molecular disulfide bond. The covalent attachment of glutathione to a protein thiol group constitutes an important redox PTM known as glutathionylation (**Figure 1**).

### ·NO-MEDIATED MODIFICATION OF PROTEIN THIOLS

·NO is a relatively unreactive gas radical and should in principle not react directly with protein thiols to form S-nitrosylated proteins. The primary cellular targets include other radical species such as $O_{2}^{\cdot -}$ to form peroxynitrite, oxygen to form ·NO_2_ and metalloproteins as those containing haems and iron–sulfur clusters. Several mechanisms of S-nitrosylation, i.e., the covalent binding of an NO group to a cysteine thiol group, have been proposed, but which one(s) operate in plant cells is not yet elucidated. S-nitrosothiols may first be formed by the reaction of ·NO with thiyl radicals generated by a one-electron oxidation of a thiolate, for example via ·NO_2_ . Alternatively, ·NO_2_ could combine first with ·NO, forming N_2_O_3_ which might subsequently react with cysteine thiolate. An additional possibility would be the transfer of haem-bound NO to a free thiol group. Once formed *de novo*, another physiologically relevant mechanism for S-nitrosothiol formation is trans-nitrosylation, i.e., the transfer of an NO moiety from a S-nitrosylated protein to another (Wang et al., 2006). Incidentally, the NO moiety can also be transferred to glutathione forming nitrosoglutathione, a possible transport and/or reservoir form (Lee et al., 2008). In support of an important role of nitrosoglutathione in plants, mutants for the nitrosoglutathione reductase gene exhibit important growth defects and modified responses to abiotic and biotic constraints (Feechan et al., 2005; Lee et al., 2008; Kwon et al., 2012).

### H_2_S-MEDIATED MODIFICATION OF PROTEIN THIOLS

The involvement of H_2_S as a signaling molecule is only emerging both in plants and animals. Like ·NO, H_2_S can play regulatory roles and modulate protein activity by binding to some protein haems. Besides, H_2_S can promote the formation of persulfide groups, a process known as S-sulfhydration, through several potential mechanisms. H_2_S could perform a nucleophilic attack on oxidized protein cysteine residues either as sulfenic acid, disulfide bond or glutathione adducts (Finkel, 2012). Another possibility is that the sulfenyl-amide intermediate, as shown for human protein tyrosine phosphatase 1B (PTP1B), can also react with H_2_S, the resulting persulfide being reduced by Trx (Krishnan et al., 2011). These reaction mechanisms are uncertain considering that H_2_S is a poor reductant compared to glutathione and that it is also less abundant and reactive. Another potential mechanism involves oxidation of H_2_S into H_2_S_2_ by reaction with ROS and subsequent nucleophilic attack by a protein thiolate. Similarly, to trans-nitrosylation, S-sulfhydryls could eventually react with another thiol, forming either a disulfide or more likely transferring its sulfur to an acceptor protein in a trans-sulfhydration reaction. Such persulfide transfer is documented in the case of the sulfurtransferase/rhodanese and cysteine desulfurase protein families (Pilon-Smits et al., 2002; Papenbrock et al., 2011).

## FUNCTIONAL SIGNIFICANCE OF REDOX PTMs

Numerous cellular processes are dependent on thiol-dependent mechanisms. Besides the purely structural role of some disulfide bonds, various effects can be ascribed to redox changes, as they can participate in catalytic, regulatory, protective and signaling mechanisms by promoting conformational changes and influencing protein–protein interactions or subcellular localization which *in fine* affect the biological activity of the modified proteins. The principal redox PTMs considered here (S-nitrosylation, S-glutathionylation, S-sulfenylation, S-sulfhydration, and disulfide bond formation) are mostly regulated by the Trx and Grx families.

An interesting and well-characterized example showing how changes in the protein oligomeric state can regulate localization is represented by the pathogen-responsive NPR1 (non-expresser of pathogenesis-related genes 1) protein. In the absence of the pathogen, NPR1 is retained in the cytosol by forming covalent disulfide-bridged oligomers. Upon reduction, NPR1 becomes monomeric and is translocated into the nucleus where it activates plant immune responses (Mou et al., 2003). While S-nitrosylation of NPR1 is likely involved in this oligomerization change, depending on the physiological context, it could either promote cytosolic retention (Tada et al., 2008) or nuclear translocation (Lindermayr et al., 2010). Other well-known examples in plants of disulfide-regulated proteins concern the dark/light-dependent oxidation/reduction of critical disulfide bonds in several enzymes of the carbon metabolism including many enzymes of the Calvin–Benson cycle (Chibani et al., 2010). By definition, the so-called regulatory cysteines are not involved in catalysis but owing to their strategic position, they modify active site access or surfaces involved in protein–protein or protein–DNA interactions.

In terms of catalysis, several enzyme families, as cysteine proteases, phosphatases, antioxidant enzymes such as Prxs, glutathione peroxidases and methionine sulfoxide reductases and many oxidoreductases, use critical reactive cysteines during the catalytic act. The functioning and regeneration of some Prxs is an interesting example as it involves the formation of several cysteine oxidation forms that divert the catalytic role into H_2_O_2_ signaling functions. The first step of the catalytic cycle is the formation of a sulfenic acid. For isoforms possessing only the peroxidatic cysteine, this species is likely reduced by the glutathione/Grxs couple (Noguera-Mazon et al., 2006). For enzymes possessing a recycling cysteine, the formation of a sulfenic acid constitutes an intermediate for the formation of a disulfide usually reducible by Trxs (Chae et al., 1994). For the so-called 2-Cys Prx subgroup, two sequence motifs surrounding the active site and specific to sensitive eukaryote isoforms, were shown to delay the formation of the intermolecular disulfide which allows, depending on H_2_O_2_ concentrations, the formation of a SO_2_H. It is now accepted that this overoxidation leads to the transient inactivation of the peroxidase activity allowing the local accumulation of H_2_O_2_ which can then promote appropriate signaling pathways (Wood et al., 2003; D’Autreaux and Toledano, 2007). While SO_2_H are mostly irreversible oxidation forms, an enzyme called sulfiredoxin (Srx) is able to catalyze the ATP-dependent reduction of the SO_2_H formed on these sensitive 2-Cys Prxs (Biteau et al., 2003). This mechanism is likely valid for plants as a Srx, dual-targeted to chloroplasts and mitochondria, can specifically reduce SO_2_H formed on 2-Cys Prxs but also on plant PrxII or mammalian PrxV (Rey et al., 2007; Iglesias-Baena et al., 2010, 2011). Interestingly, the SO_2_H reduction proceeds via the formation of a phosphoryl intermediate on the sulfinyl moiety, attacked by the catalytic cysteine of Srx finally forming a thiosulfinate intermediate between Prx and Srx (Jonsson et al., 2008; Roussel et al., 2008).

To further illustrate cysteine-based signaling mechanisms in response to peroxides, it is worth mentioning that the DNA binding activity of numerous transcription factors in microbes (bacteria or fungi), such as OxyR, OhrR, AP1, or CrtJ is regulated by the primary formation of a sulfenic acid which is often transformed into a disulfide bond (D’Autreaux and Toledano, 2007; Cheng et al., 2012). To date, few examples of such signaling functions are known in plants.

The biochemical characterization of recombinant proteins coupled to redox proteome studies often led to the observation that a single protein, either a metabolic enzyme or a signaling protein can undergo a plethora of redox PTMs. In this context, it is not trivial to differentiate between protective and signaling purposes. This is well exemplified in mammals for glyceraldehyde-3-phosphate dehydrogenase (GAPDH), PTP1B or the p65 subunit of the NF-kB transcription factor. In these proteins, the critical cysteine can react with H_2_O_2_, ·NO and H_2_S derived forms, forming sulfenic acid, nitrosothiol or persulfide intermediates, respectively, that can eventually form sulfenyl-amide or glutathione adduct or interchange with each other. Whereas most of these reversible modifications lead to protein inactivation, S-sulfhydration can be distinct since it usually activates the targeted proteins as shown for GAPDH and p65 (Kelleher et al., 2007; Mustafa et al., 2009; Krishnan et al., 2011; Paul and Snyder, 2012).

All these observations suggest that there may be biological differences for each of these different PTMs and possibly hierarchies. For instance, it was shown that sulfhydration of Cys38 in p65, which stimulates its transcriptional activity, predominated early after an H_2_S-forming TNF-α treatment but that it declined with time being succeeded by a reciprocal enhancement of Cys38 nitrosylation which inhibited it (Kelleher et al., 2007; Sen et al., 2012). Although the physiological consequence and significance of multiple redox PTMs is often less understood in plants, redox proteomic studies already confirmed that many proteins can be either nitrosylated, or glutathionylated or disulfide-bonded.

## PROTEOMIC IDENTIFICATION OF REDOX PTMs

While the identification of reactive cysteines is crucial for understanding protein function and regulation, there is no universal signature allowing their identification. Analyses based on the strict conservation of cysteines between homologous proteins (in particular in CxxC/S motifs), or their replacement by selenocysteines in orthologs found in some organisms, often proved to be valuable. However, it neither provides an exhaustive list of proteins containing these reactive cysteines nor indicates which redox PTMs exist in a cellular context and how large proportion of a reactive cysteine is modified. Thus, besides computational approaches and biochemical approaches using purified recombinant proteins, many gel-based or gel-free wide-scale proteomic approaches have been designed to identify reactive cysteines and associated redox PTMs from complex protein extracts.

A direct proteomic method to detect proteins with disulfide bonds and called Redox 2D-PAGE or diagonal SDS-PAGE is based on the differential migration of proteins containing intra-or intermolecular disulfides under non-reducing (first dimension) and reducing (second dimension) conditions (Cumming, 2008). In this second dimension, proteins without disulfide bonds will lie in a diagonal line on the 2D gel whereas proteins with inter- or intramolecular disulfide bonds will migrate below or above this diagonal. Alternatively, initially assuming that disulfide bonds are preferentially reduced by Trxs, the identification of Trx targets was thought to constitute a good representative, although non-exhaustive, list of disulfide bond-containing proteins. Several hundreds of putative plant Trx target proteins, listed in (Montrichard et al., 2009), have been isolated using affinity chromatography columns where mutated Trxs were immobilized in order to freeze a covalent interaction with their targets or using thiol labeling after Trx reduction (Yano et al., 2001). However, with the observation that some Trxs can reduce sulfenic acids, S-nitrosothiols, glutathionylated and sulfhydrated cysteines and could promote trans-nitrosylation reactions, this approach may have detected most reversible redox PTMs (Mitchell and Marletta, 2005; Benhar et al., 2008; Krishnan et al., 2011; Bedhomme et al., 2012).

**FIGURE 2:**
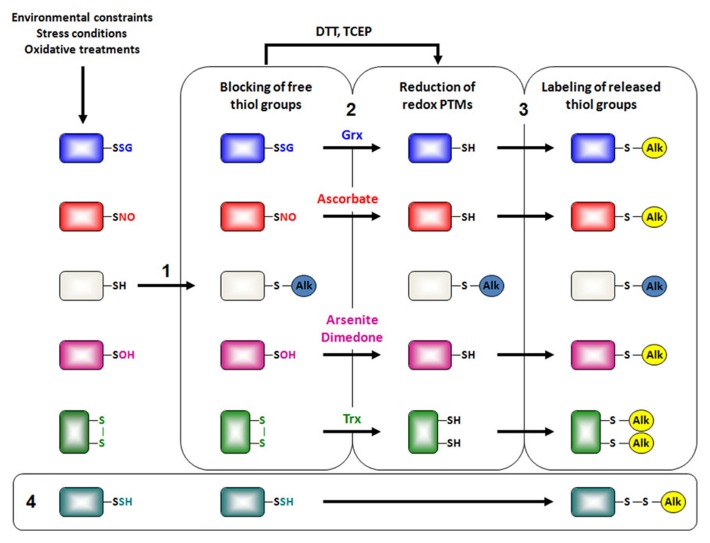
**Indirect chemical detection of redox PTMs by proteomics.** Besides the wish for detecting basal redox PTMs, experiments are usually designed to assess *in vivo* redox changes after applying an oxidative stress treatment. Most current approaches to detect redox PTMs rely on the same three-step strategy. The first step consists of blocking free thiol groups with alkylating agents such as N-ethylmaleimide (NEM), iodoacetamide (IAM) and its isotopically light ^12^C- derivative or methyl methanethiosulfonate (MMTS) (1). The second step consists of the reduction of reversibly oxidized cysteine residues (2). General reductants as dithiothreitol (DTT) or Tris(2-carboxyethyl)phosphine hydrochloride (TCEP) are used to identify all types of oxidized cysteines. In contrast, selective chemical agents (ascorbate, arsenite, dimedone) or enzymes (glutaredoxins, thioredoxins) are used for the reduction of specific redox PTMs. Finally, the third step corresponds to the labeling of liberated thiol groups by reaction with biotinylated-, fluorescent- or isotopically heavy ^13^C-derivatives of alkylating reagents mentioned previously, keeping in mind that fluorescent reagents are rather devoted to the detection of modified proteins, whereas biotinylated reagents are devoted to protein enrichment for subsequent mass spectrometry analyses (3). Readers interested by the qualitative ICAT approach can obtain more details in a recent review (Leonard and Carroll, 2011). Interestingly, because MMTS does not seem to reduce persulfide groups, Mustafa et al. (2009) demonstrated that these persulfide groups could be detected without the reduction step if MMTS is used for the first alkylation step (4).

Most of the current methods designed to detect reactive cysteines and redox PTMs are indirect and are based on the differential alkylation of reduced and oxidized thiols (**Figure 2**). They require an initial step of thiol alkylation of free unreactive cysteines using generally N-ethylmaleimide (NEM), iodoacetamide (IAM) for irreversible modifications or MMTS (methyl methanethiosulfonate) for reversible modifications. In a second step, the various types of PTM are reduced by general tris(2-carboxyethyl)phosphine, dithiothreitol (TCEP, DTT) or specific chemical compounds or enzymes. Finally, nascent thiols are labeled by derivation with biotinylated- or fluorescent- forms of these alkylating reagents. The biotinylated proteins are recovered on avidin columns and identified preferentially using a gel-free method by LC-MS-MS. Until recently, this was rather a qualitative inventory but quantitative thiol-trapping techniques (namely OxICAT, isotope-coded affinity tag for the identification of Oxidized cysteines; isoTOP-ABPP, isotopic tandem orthogonal proteolysis–activity-based protein profiling), essentially based on the use of isotopic light ^12^C- and heavy ^13^C-forms of IAM, have been developed to identify the site(s) of modifications and assess the degree of modification and reactivity (Hagglund et al., 2008; Leichert et al., 2008; Marino et al., 2010; Weerapana et al., 2010).

For sulfenic acids, reduction was initially achieved through arsenite but other probes derived from dimedone, namely DAz-2 and DYn-2, have recently been engineered (Leonard and Carroll, 2011; Roos and Messens, 2011). For S-glutathionylated and S-nitrosylated proteins, reduction is usually performed using Grxs and ascorbate, respectively. The latter strategy was called biotin switch method (Jaffrey and Snyder, 2001). For instance, a modified biotin switch omitting ascorbate was used for isolating S-sulfhydrated proteins (Mustafa et al., 2009). Some alternative methods for assessing redox PTMs or recent improvement of these differential alkylation methods can be found in (Gao et al., 2009; Wang and Xian, 2011; Astier and Lindermayr, 2012). The proteins identified through the different methods show only partial overlap, indicating that a significant portion of modifications are specific, being dependent on the protein local environment and/or on the applied oxidizing conditions. Long lists of plant proteins undergoing various redox PTMs, especially glutathionylation and nitrosylation, and cellular processes affected were recently published (Gao et al., 2009; Astier et al., 2012; Paul and Snyder, 2012; Zaffagnini et al., 2012).

All these methods are usually subject to the same limitations. Alkylation, reduction and labeling are usually performed on cell lysates and though protein extraction is performed using acidic or sometimes anaerobic conditions, it does not entirely preclude that modifications of the cysteine redox state occur during the procedure or that the modifications are insufficiently trapped. To circumvent this problem, cell permeable probes were developed, in particular for sulfenic acids (Leonard and Carroll, 2011). As explained above, another major drawback of such studies concerns the specificity of a given reductant for a given PTM as also discussed for S-nitrosothiol in (Wang and Xian, 2011). This will clearly need to be addressed in the future. Moreover, protein abundance is often a limiting factor, mostly for gel-based methods. Possibilities to circumvent this problem are to perform pre-fractionation or to use biotin affinity for avidin in order to increase the amount of modified proteins and decrease complexity of the sample. Another very important question that often remains unanswered is to determine the site of modification and to what extent a given cysteine is modified. Answering this question requires ICAT-derived gel-free strategies that were used only in rare cases (Hagglund et al., 2008; Leichert et al., 2008).

## Conflict of Interest Statement

The authors declare that the research was conducted in the absence of any commercial or financial relationships that could be construed as a potential conflict of interest.
